# Evaluation of HIV testing algorithms in Ethiopia: the role of the tie-breaker algorithm and weakly reacting test lines in contributing to a high rate of false positive HIV diagnoses

**DOI:** 10.1186/s12879-015-0769-3

**Published:** 2015-02-03

**Authors:** Leslie Shanks, M Ruby Siddiqui, Jarmila Kliescikova, Neil Pearce, Cono Ariti, Libsework Muluneh, Erwan Pirou, Koert Ritmeijer, Johnson Masiga, Almaz Abebe

**Affiliations:** Médecins Sans Frontières, Amsterdam, The Netherlands; Médecins Sans Frontières, London, UK; London School of Hygiene and Tropical Medicine, London, UK; Ethiopian Health and Nutrition Research Institute, Addis Ababa, Ethiopia

**Keywords:** HIV, Rapid diagnostic test, Confirmation test, False positive, Ethiopia, Médecins Sans Frontières

## Abstract

**Background:**

In Ethiopia a tiebreaker algorithm using 3 rapid diagnostic tests (RDTs) in series is used to diagnose HIV. Discordant results between the first 2 RDTs are resolved by a third ‘tiebreaker’ RDT. Médecins Sans Frontières uses an alternate serial algorithm of 2 RDTs followed by a confirmation test for all double positive RDT results. The primary objective was to compare the performance of the tiebreaker algorithm with a serial algorithm, and to evaluate the addition of a confirmation test to both algorithms. A secondary objective looked at the positive predictive value (PPV) of weakly reactive test lines.

**Methods:**

The study was conducted in two HIV testing sites in Ethiopia. Study participants were recruited sequentially until 200 positive samples were reached. Each sample was re-tested in the laboratory on the 3 RDTs and on a simple to use confirmation test, the Orgenics Immunocomb Combfirm® (OIC). The gold standard test was the Western Blot, with indeterminate results resolved by PCR testing.

**Results:**

2620 subjects were included with a HIV prevalence of 7.7%. Each of the 3 RDTs had an individual specificity of at least 99%. The serial algorithm with 2 RDTs had a single false positive result (1 out of 204) to give a PPV of 99.5% (95% CI 97.3%-100%). The tiebreaker algorithm resulted in 16 false positive results (PPV 92.7%, 95% CI: 88.4%-95.8%). Adding the OIC confirmation test to either algorithm eliminated the false positives. All the false positives had at least one weakly reactive test line in the algorithm. The PPV of weakly reacting RDTs was significantly lower than those with strongly positive test lines.

**Conclusion:**

The risk of false positive HIV diagnosis in a tiebreaker algorithm is significant. We recommend abandoning the tie-breaker algorithm in favour of WHO recommended serial or parallel algorithms, interpreting weakly reactive test lines as indeterminate results requiring further testing except in the setting of blood transfusion, and most importantly, adding a confirmation test to the RDT algorithm. It is now time to focus research efforts on how best to translate this knowledge into practice at the field level.

**Trial registration:**

Clinical Trial registration #: NCT01716299

## Background

Ethiopia has a HIV prevalence of 1.3% (95% CI 1.2%-1.5%) in the adult population [1]. Considerable progress has been made over the last decade in scaling up access to testing across the country. In 2011, the number of people accessing HIV testing reached close to 10 million [2]. Scale up of HIV testing is possible due to diagnostic algorithms that employ HIV rapid diagnostic tests (RDTs). In Ethiopia, a tiebreaker regimen consisting of 3 RDTs in series is the national algorithm chosen after a thorough evaluation period. It uses HIV (1 + 2) Antibody Colloidal Gold (KHB, Shanghai Kehua Bio-engineering Co Ltd, China) as a screening test, followed by HIV 1/2 STAT-PAK® (Chembio Diagnostics, USA) if positive. Where the result of STAT-PAK® is discordant with KHB, a third test, Unigold™ HIV (Trinity Biotech, Ireland), is used as a tiebreaker to determine the result.

Rapid diagnostic tests are essential tools to screen for HIV, and are designed for use with confirmatory tests such as Western Blot to diagnose infection. However given resource constraints, WHO has developed testing guidelines that use 2–3 RDTs together in an algorithm to diagnose HIV [3]. While these algorithms allow decentralisation of HIV testing and scale up of access, they can come with the compromise of false positive results. This risk is described in a number of different studies [4-8] and has been shown to vary geographically and over time [9]. The risk of false positive results is linked to cross-reactivity, whereby non-HIV antibodies or protein in the blood falsely react with the antigens of the HIV RDTs. Where both RDTs cross react, a false positive diagnosis will result.

A number of studies have shown a link between false positive results and a weaker than usual test line on the RDT [4,5,8,10-13]. However current manufacturers’ recommendations are that any colour of the test line is interpreted as positive, which can result in misdiagnosis of HIV if a second test is also positive.

Médecins Sans Frontières-Operational Centre Amsterdam (MSF) opened a project in Humera to support the Tigray Regional Health Bureau with diagnosing and treating visceral leishmaniasis (VL) in 1997. HIV testing activities started the following year, with anti-retroviral treatment (ART) available from 2004. In 2008, MSF handed over the project to the Health Bureau. MSF has worked in Abdurafi providing diagnosis and treatment for VL and HIV since 2004. In both sites, health care staff and patients identified concerns about possible misclassification of HIV infection. In response, MSF introduced an alternate algorithm in Abdurafi, using two RDTs in series, followed by a confirmation test. The confirmation test used, the Orgenics Immunocomb® II, HIV 1&2 Combfirm (OIC), separately detects the p24, p31, gp41, gp120, and gp36 antibodies. It is performed by peripheral laboratory staff and produces results in two hours at a cost of $5 per test. The OIC in the first 15 months of use in Abdurafi identified 7.1% of positive results on a serial algorithm of Determine HIV-1/2 (Abbott Laboratories) and Unigold™ to be false positive reactions [14]. However the OIC is not part of the national algorithm, and there is little published on its performance characteristics.

We designed a study using standard WHO methodology to evaluate the performance of different RDT algorithms including the addition of the OIC confirmation test. We included a secondary objective on the predictive value of weak positive test lines. Two other objectives looking at the potential association between VL infection and false positive results and a novel method of confirmation testing, are reported separately.

## Methods

### Setting

The study site was in a MSF supported health centre in Abdurafi and a zonal hospital in Humera. The populations included residents as well as high numbers of migrant workers who are present seasonally. Testing took place in the designated counselling and testing (CT) centres in each site, in addition to the antenatal clinic, hospital and outpatient department in Humera.

### Inclusion criteria

All clients, aged > = 5 years, presenting to be tested for HIV in the study sites were invited to participate in the study. Study participants underwent informed consent procedures and had a written consent form signed by the participant or the guardian.

### Sample size

A sample size of 200 serial algorithm positive and 200 algorithm negative participants was chosen based on the WHO guidance for evaluation of RDTs [15]. To achieve the sample, all KHB positive samples were included along with every 10th KHB negative sample until a minimum of 200 positive samples were reached.

#### Information collected

Information recorded included age, sex, village, residency status (migrant worker, settler, resident, commercial sex worker, other), reason for testing, and time of most recent risk exposure. Clinical information recorded included CD4 count and presence/absence of active VL infection.

#### Testing

Initial testing was done on whole blood using the KHB-STAT/PAK®/Unigold™ tiebreaker algorithm in Humera, and on plasma using the KHB/STAT-PAK® serial algorithm in Abdurafi. In the laboratory, samples were tested with 3 RDTs (KHB, STAT-PAK®, and Unigold™) on whole blood and plasma. Laboratory technicians were blinded to the CT results. All tests were performed according to manufacturer’s instructions. Invalid tests where the control line did not appear were discarded and repeated on new test devices according to manufacturers’ instructions.

Tests were interpreted as positive when there was any colouration of the test line. Positive results were further classified as weak positive when the test line was significantly thinner and weaker than normal. A photo card was developed to guide interpretation.

All samples underwent testing by OIC and Western Blot with technicians blinded to earlier results. OIC was performed at each study site while the Western Blots were all performed at the MSF laboratory in Abdurafi. Interpretation of OIC was based on the stricter criteria employed in the *Agence Nationale d’Accréditation et d’Evaluation en Santé* guidelines [16]. Three to four spots were considered positive, two as indeterminate, and zero or one spot as negative. Gp36 was not included in determining the number of reactions.

Western Blot (WB) testing was performed using MP Diagnostics HIV Blot 2.2 at the MSF Abdurafi laboratory. Interpretation of results was based on American Red Cross recommendations [17].

Samples indeterminate on WB or OIC were repeated and if still indeterminate, underwent DNA PCR examination using Roche Amplicor DNA v1.5 on dry blood spots (DBS) at the Ethiopian Health and Nutrition Research Institute (EHNRI) laboratory based in Addis Ababa, allowing diagnosis of subtypes A-D. The Global Clinical and Viral Laboratory in South Africa provided quality control for PCR and confirmed results using Cobas® AmpliPrep/Cobas® TaqMan® HIV-1 Qual test which detects HIV subtypes A to H. Where results between the two labs were discrepant, the result from South Africa was used.

The final gold standard result was that of the Western Blot, and where Western Blot was indeterminate, the PCR result.

#### Quality control

All staff underwent training on the study standard operating procedures by the MSF laboratory supervisor, and received regular monitoring and supervision. As previously described all RDT results were controlled at the laboratory, and discrepant results repeated.

#### Analysis

The RDT result on plasma was considered the final result for the purposes of the analysis. As the samples received all 3 RDTs in the laboratory, each sample regardless of the initial algorithm was evaluated for both the serial and tiebreaker algorithm. It was also possible to calculate an alternate algorithm to give results for a serial KHB/Unigold™ algorithm, and a KHB/Unigold™ /STAT-PAK® tiebreaker algorithm. Discordant test results (one RDT positive, the other negative) were classified as negative, and indeterminate OIC results as positive for the calculation of predictive values and sensitivity/specificity.

Predictive values and sensitivity and specificity were estimated from the 2 × 2 table of observed results after weighting based on the sampling proportion of the KHB positive and negative samples. Confidence intervals for each of the test parameters were calculated using exact binomial intervals. PPVs of RDT algorithms from the main sample were compared using an analogue of McNemar’s statistic (Z score) derived from a marginal logistic regression model [18]. Where the logistic regression model was unable to give a score a bias-corrected bootstrap was employed. Fisher’s exact test was used to compare categorical variables for the false positive analysis with the Mann–Whitney test for continuous variables.

Statistical analysis was done using Stata version 12 (StataCorp, Texas, USA).

#### Ethical review

The study received approval from the MSF Ethics Review Board, the EHNRI Research and Ethical Clearance Committee, and the National Research Ethics Review Committee, Ministry of Science and Technology in Ethiopia.

## Results

2622 individuals were screened from December 2010 to July 2011, 1297 (59.2%) in Humera and 895 (40.8%) in Abdurafi. 430 individuals were eligible for analysis, representing all KHB positives and 10% of KHB negatives. One sample was excluded due to missing WB and PCR results, and another was excluded due to a duplicate identity number. This resulted in a total sample of 428. HIV prevalence was 7.7% (203/2620). A description of the demographics of the study participants is found in Table 1.

**Table 1:** **Demographics of study participants**

		**Humera site**	**Abdurafi site**	**Total**
		**N (%)**	**N (%)**	**N (%)**
Number		230	198	428
Age	Mean [range] in years	31.3 [10–65]	28.0 [10–67]	29.7 [10–67]
Sex	Male	140 (61.1)	119 (60.1)	259(61.0)
Residential status	Resident	122 (53.3)	101 (51.0)	223 (52.3)
	Migrant	47 (20.5)	68 (34.3)	115 (26.9)
	Settler	11 (4.8)	19 (9.6)	30 (7.0)
	Other	49 (21.4)	10 (5.1)	59 (13.8)
Reason for testing	Diagnostic testing	65 (28.4)	56 (28.3)	121 (28.3)
	Symptomatic	80 (35.0)	16 (8.1)	96 (22.5)
	Curious about status	8 (3.5)	63 (31.8)	71 (16.6)
	Pre-marriage	15 (6.6)	22 (11.1)	37 (8.7)
	Other	61 (26.6)	41 (20.7)	102 (23.9)

The WB identified 201 positives, 166 negatives and 61 indeterminates (59 of which were negative on PCR and 2 positive). The OIC identified 198 positives, 223 negatives and 7 indeterminates (5 positive on PCR and 2 negative). There were no positive HIV-2 results on either the OIC or WB.

Test performance measures of the individual RDTs are in Table 2. Each RDT had a specificity of 99% or greater.

**Table 2:** **Sensitivity, specificity and predictive values of individual RDTs and the OIC confirmation test (N = 2620)**

**RDT**	**Results (95% Confidence interval)**			
	**Sensitivity**	**Specificity**	**PPV**	**NPV**
KHB	100% (98.2-100)	99.1% (98.6-99.4)	89.8% (85.1-93.4)	100% (99.8-100)
Unigold	99.0% (96.5-99.9)	99.0% (98.5-99.4)	89.3% (84.5-93.0)	99.9% (99.7-100)
STAT-PAK	100% (98.2-100)	99.9% (99.7-100)	98.5% (95.8-99.7)	99.8% (99.9-100)
**Confirmation Test**				
OIC*	100% (98.2-100)	99.1% (96.8-99.9)	99.0% (96.5-99.9)	100% (98.4-100)

*OIC indeterminate results were classified as positive for the purpose of the analysis, however in practice, OIC indeterminate results require further testing and cannot be used to diagnose HIV.

### Serial Algorithms

The KHB/STAT-PAK® serial algorithm had one false positive result and no false negatives. There were 22 discordant results, all of which were resolved negative by WB and PCR.

The addition of the OIC confirmatory test to the KHB/STATPAK® algorithm eliminated the false positive result. There was no statistically significant difference in PPV between the serial KHB/STAT-PAK® algorithm and the corresponding serial OIC algorithm (p = 0.33).

An alternate serial algorithm with KHB/Unigold™ resulted in 16 false positives, 0 false negatives, and 9 discordants, 7 (77.8%) of which were resolved to be negative. The addition of the OIC test to the algorithm significantly improved the PPV (p = 0.004). Details are in Tables 3 and 4.

**Table 3:** **All samples recruited for the initial sample, N = 428**

**Algorithm**	**N (%)**	**Gold Standard result**	
		**Positive (%)**	**Negative (%)**
Serial KHB/STAT-PAK			
Positive	198	198 (100%)	0
Weak positive	6	5 (83.3%)	1 (16.7%)
Negative	202	0	202 (100%)
Discordant*	22	0	22 (100%)
Serial KHB/STAT-PAK-OIC			
Positive	198	198	0
Negative	203	0	203
Indeterminate	5	5 (100%)	0
Discordant*	22	0	22 (100%)
Tiebreaker: KHB/STAT-PAK/Unigold			
Positive	198	198	0
Weak positive	21	5 (23.8%)	16 (76.2%)
Negative	209	0	209
Tiebreaker KHB/STAT-PAK/Unigold-OIC			
Positive	198	198 (100%)	0
Indeterminate	6	5 (83.3%)	1 (16.7%)
Negative	224	0	224 (100%)
Serial KHB-Unigold			
Positive	182	182	0
Weak positive	35	19 (54.3%)	16 (45.7%)
Negative	202	0	202
Discordant*	9	2 (22.2%)	7 (77.8%)
Serial KHB-Unigold-OIC			
Positive	196	196 (100%)	0
Negative	217	0	217 (100%)
Indeterminate	6	5 (83.3%)	1 (16.7%)
Discordant*	9	2 (22.2%)	7 (77.8%)
Tiebreaker: KHB-Unigold-Statpak			
Positive	184	184 (100%)	0
Weak positive	35	19 (54.3%)	16 (45.7%)
Negative	209	0	209 (100%)
Tiebreaker KHB-Unigold-Statpak-OIC			
Positive	198	198 (100%)	0
Negative	224	0	224 (100%)
Indeterminate	6	5 (83.3%)	1 (16.7%)
TOTAL	428	**203 (47.4%)**	**225 (52.6%)**

*Discordant RDT results are reported separately because in the confirmatory algorithms (eg KHB-Unigold-OIC), a serological confirmation test is only indicated if both test 1 and test 2 are positive. In practice, discordant RDT results are repeated after several weeks to rule out seroconversion.

**Table 4:** **Sensitivity, specificity and predictive values of algorithms (N = 2620)**

**Algorithm**	**Results (95% Confidence interval)**			
	**Sensitivity**	**Specificity**	**Positive predictive value**	**Negative predictive value**
Serial KHB/STAT-PAK	100%	100%	99.5% (97.3-100)	100% (98.4-100)
	(98.2-100)	(99.8-100)		
Serial	100%	100%	100% (98.2-100)	100% (98.4-100)
KHB/STAT-PAK-OIC	(98.2-100)	(98.4-100)		
Tiebreaker	100%	99.3%	92.7% (88.4-95.8)	100% (98.3-100)
KHB/STAT-PAK/Unigold	(98.2-100)	(98.9-99.6)		
Tiebreaker	100%	100%	99.5% (97.3-100)	100% (98.4-100)
KHB/STAT-PAK/Unigold-OIC	(98.2-100)	(99.8-100)		
Serial KHB/Unigold	99.0%	99.3%	92.6% (88.3-95.7)	99.9% (99.7-100)
	(96.5-99.9)	(98.9-99.6)		
Serial KHB/Unigold-OIC	99.0%	100%	99.5% (97.3-100)	99.9% (99.7-100)
	(96.5-99.9)	(99.8-100)		
Tiebreaker	100%	99.3%	92.7% (88.4-95.8)	100% (98.3-100)
KHB/Unigold/STAT-PAK	(98.2-100)	(98.9-99.6)		
Tiebreaker	99.0%	100%	99.5% (97.3-100)	99.9% (99.7-100)
KHB/Unigold/STAT-PAK-OIC	(96.5-99.9)	(99.8-100)		

### Tiebreaker algorithm

The KHB/STAT-PAK®/Unigold™ tiebreaker algorithm yielded 16 false positive results and 0 false negatives as shown in Table 3. Addition of the OIC test to the algorithm eliminated the false positive results and added 6 indeterminate results, 5 of which were resolved positive. The PPV of the OIC algorithm was significantly improved compared to the tiebreaker alone (p < 0.001).

An alternate tiebreaker algorithm of KHB/Unigold™/STAT-PAK® also resulted in 16 false positives and no false negatives. Addition of the OIC test eliminated the false positives and added 6 indeterminate results. Five of the indeterminates were resolved positive. The OIC test significantly improved the PPV compared with the alternate tiebreaker algorithm alone (p < 0.001).

Compared to the serial KHB/STAT-PAK® algorithm, the tiebreaker did significantly worse (p = 0.004). Looking at the alternate algorithm, the difference between the serial KHB/Unigold™ and the corresponding tiebreaker, was not significant (p = 0.16).

Details are in Table 4.

### Weak positives

51 RDT results were recorded as weak positive on whole blood in the laboratory: 22 (N = 411) KHB, 8 STAT-PAK® (N = 411), and 21Unigold™ (N = 91). On a total of 428 plasma samples, there were 64 weak reactions: 21 KHB, 7 STAT-PAK®, and 36 Unigold™. The proportion of weak positives amongst the total positive reactions in whole blood versus plasma was not found to be significantly different with p values of 0.745, 0.79 and 0.82 for KHB, STAT-PAK®, and Unigold™ respectively.

The kappa statistic for inter-reader agreement between the CT and the laboratory in Humera for weak versus strong positives on whole blood was 0.85 (p < 0.001) for KHB and 0.32 (p < 0.001) for STAT-PAK®. The kappa statistic for inter-reader agreement between the CT and the laboratory in Abdurafi for weak versus strong positives on plasma was 0.79 (p < 0.001) for KHB and 0.49 (p < 0.001) for STAT-PAK®.

The KHB/STAT-PAK® serial algorithm had 6 weak positives, 1 (17.7%) was false positive. The KHB/STAT-PAK®/Unigold™ tiebreaker algorithm had 21 weak positive results, 16 (76.2%) were false positives. Both the serial KHB/Unigold™ and the tiebreaker KHB/Unigold™/STAT-PAK® algorithms had 35 weak positives. 16 (45.7%) were false positive. All false positives results, regardless of algorithm, had at least one RDT result classified as weak positive.

Removing the weak positives from the algorithms, significantly improved the performance of the tiebreaker algorithm (p < 0.001) but not the serial algorithm (p = 0.30) respectively (Table 5).

**Table 5:** **Performance characteristics of algorithms with weak positives excluded**

**Algorithm**	**(95% Confidence interval)**				
	**Sensitivity**	**Specificity**	**Positive predictive value**	**Negative predictive value**	**HIV prevalence**
Serial KHB/STAT-PAK	100%	100%	100%	100%	7.59%
	(98.2-100)	(98.4-100)	(98.2-100)	(98.4-100)	
Tiebreaker	100%	100%	100%	100%	7.59%
KHB/STAT-PAK/Unigold	(98.2-100)	(98.3-100)	(98.2-100)	(98.3-100)	
Serial KHB/Unigold	98.9%	100%	100%	99.9%	7.06%
	(96.1-99.9)	(98.3-100)	(98.0-100)	(99.7-100)	
Tiebreaker	100%	100%	100%	100%	7.06%
KHB/Unigold/STAT-PAK	(98.0-100)	(98.3-100)	(98.0-100)	(98.3-100)	

The PPV of a single weak positive RDT result versus that of a strong positive is found in Table 6.

**Table 6:** **Positive predictive value of weak RDT result versus strong positive on plasma**

**Test**	**Weak positive (95% CI)**	**Strong positive (95% CI)**
KHB	9.5% (1.2-30.4)	98.1% (95.3-99.5)
Unigold	50.0% (32.9-67.1)	96.8% (93.2-98.8)
STAT-PAK	57.1% (18.4-90.1)	100%(98.2-100)

### Description of false positives

A total of 16 individuals were identified on the tiebreaker algorithm as false positives (FP). Table 7 contains a breakdown of the characteristics of the false positive samples.

**Table 7:** **Characteristics of false positives compared with true positives**

**Variable**		**False positives**	**True positives**	**p value**
		**N (%)**	**N (%)**	
Age	Average	29 years	32 years	p = 0.041*
Sex	Male	16 (100)	111 (54.7)	p = 0.000*
	Female	0	92 (45.3)	
Site	Humera	4 (25.0)	134 (66.0)	p = 0.002*
	Abdurafi	12 (75.0)	113 (34.0)	
Residence status	Migrant	6 (37.5)	60 (29.6)	p = 0.339
	Settler	0	18 (8.8)	
	Resident	10 (62.5)	89 (43.8)	
	Commercial sex worker	0	23 (11.3)	
	Other	0	13 (6.4)	
Date of visit**	Mean days	122	176	p = 0.025*
CD4	Median	491[107–992]	271 [8–1051]	p = 0.010*
	[range]			
Visceral Leishmaniasis	Positive	2 (12.5)	3 (1.5)	p = 0.044*
	Negative	14 (87.5)	200 (98.5)	

*Significant at an alpha level of 0.05.

**Days from date of first recruitment.

The OIC results for the 16 false positives were: 13 samples: 0 reaction; 2 samples: 1 reaction; and 1 sample: 2 reactions. The cross-reacting antibodies on OIC were p24 (3), and p31 (1). Western Blot on the same samples had 5 negatives (zero or p17 only), and 11 indeterminate. Individual bands detected on WB indeterminate samples were: p24 (11), p17 (1), and p51 (1). All the OIC p24 positives were also detected on the WB.

## Discussion

Our results demonstrate that the current Ethiopian algorithm, a 3 RDT tiebreaker algorithm, has a high proportion of false positives (7.7%) in our study population with a HIV prevalence of 7.7%. There were 16 individuals falsely identified as HIV infected. Altering the algorithm, such that the final tiebreaker RDT would be STAT-PAK® rather than Unigold™ did not improve the performance. The 3 RDTs, all exceeded the WHO criteria for specificity (>98%) yet did not achieve the target PPV for the algorithm of >99% [3]. This suggests that it is the choice of algorithm rather than a poorly performing RDT that is responsible for the high percentage of false positives.

Similar poor performances of the tiebreaker have been reported elsewhere. A study from the Rakai cohort in Uganda, reports a false positive proportion of 43.7% (129/295) with a tiebreaker algorithm of Determine/STAT-PAK®/Unigold™ at a HIV prevalence of 11.2% [4]. A separate Ugandan study in a higher prevalence population looked at the performance of the tiebreaker when two out of three tests were positive. 14 of 29 (48.2%) were confirmed negative on DNA PCR [19]. In a large study conducted in Lusaka, Zambia and Kigali, Rwanda, samples with 2 out of 3 RDTs positive were found to be negative for HIV infection in 17 out of 37 (46%) of cases [20].

Many authorities mistakenly assume the tiebreaker is a WHO recommended algorithm. In fact, the WHO recommends serial or parallel testing with 2 RDTs for high prevalence populations (>5%), and 3 tests in series for low prevalence populations (<5%). Positive results should only be given if all 3 tests are positive. Those with 2 out 3 tests positive are advised they need further testing [3]. The discordant result between two RDTs that triggers the use of the third RDT is an indicator that cross reactivity is occurring. In our sample, 100% of discordant results on the serial KHB/STAT-PAK® algorithm were resolved as negative as were 77.8% of the discordants on the alternate KHB/Unigold™ algorithm. In the setting of discordant results between 2 RDTs, a confirmatory test is needed because a third RDT will be vulnerable to similar cross-reactivity.

The KHB/STAT-PAK® serial algorithm yielded a single false positive result. When the serial algorithm was changed to KHB/Unigold™, the results were markedly worse and similar to the tiebreaker regimen due to the poor performance of Unigold™. The addition of the OIC confirmation test to either the serial or the tiebreaker algorithm eliminated all the false positive results. In 3 of the 4 algorithms tested, it significantly improved the PPV compared to no confirmation test. The exception was the serial KHB/STAT-PAK® algorithm which performed adequately without a confirmation test. However the addition of the OIC did identify one individual who otherwise would have been falsely labelled as HIV positive.

It is important to state that these results were obtained with a stricter interpretation of OIC, as described in the methodology section. The results confirmed this choice; there were no misclassifications of positive or negative results on OIC compared to the gold standard. A drawback to the OIC is that similar to other serological confirmation tests, there were indeterminate tests for which a result could not be given on the same day. There were 6 algorithm positive results that were indeterminate on OIC for the tiebreaker versus 13 on the Western Blot alone. Looking at the serial algorithm, there were 5 OIC indeterminate results versus 3 for Western Blot. In calculating the performance of the OIC algorithms, we classified these results as positive. If the indeterminate algorithm results are excluded from the sample, then the sensitivity, specificity and predictive values of the confirmation algorithms are all 100%. In practice patients with indeterminate results are told their tests are inconclusive, and asked to come back for further testing. When the result is not clarified on repeat testing, then often a PCR is needed, as the Western Blot is likely to show the same indeterminate result.

Finally, the OIC is one type of confirmation test that is suitable for use in peripheral laboratories. There is an urgent research need to develop and evaluate other simple confirmation tests, which are affordable, cold-chain independent, and can be performed at peripheral level.

### Description of false positives

There were 5 characteristics that were significantly associated with false positive results on univariate analysis: age, male sex, active VL infection, the Abdurafi study site and the time of enrolment in the study. It was not possible to do multivariate analysis in order to explore these associations further due to small sample size. It is therefore difficult to conclude on the clinical significance of these findings.

The commonest band present on the false positives samples was p24, as 61.1% of FPs were positive for p24 by WB and 16.7% by OIC. This suggests that much of the cross-reactivity responsible for the falsely positive RDTs may be due to p24 and is consistent with previous findings from Ethiopia [21]. Antibody to p24 antigen is one of the earliest antibodies to appear, therefore raising the possibility that our false positives were seroconverting. However the fact that all of these cases had negative PCR testing indicates that this is cross-reactivity rather than early seroconversion.

### Weakly reactive test lines

All of the false positive results in this review resulted from weak positive RDT results. Excluding weak positives from the analysis results in a 100% PPV for all the algorithms studied. This contrasts sharply with the PPV of the weak positive tiebreaker algorithm currently in use in Ethiopia, which was found to be just 23.8% (95% CI 8.2-47.2). All 3 RDTs demonstrated weak positivity, though STAT-PAK® had fewer weak lines than Unigold™ and KHB. This is the first report of which we are aware reporting KHB weak positives. There is evidence of Determine, STAT-PAK®, and Unigold™ showing this phenomenon in multiple countries which suggests this is a class effect rather than one specific to a particular RDT or geographic location [4,5,8,10-13]. One report suggests that weak positive results are more frequent with plasma versus whole blood [11]. Our results do not support this finding.

The poor specificity of weak positive test lines is consistent with that found by other researchers and is felt to reflect the occurrence of cross reactivity. Our results further reinforce the recommendation of a growing body of researchers that weak positive test lines should be interpreted as indeterminate, and require further testing before giving a result [4,5,8,11,12]. The exception is in the setting of blood transfusions where any colouration of the test line should be read as positive. Table 8 provides a summary of the data used to support these recommendations. Given this body of evidence, it is now time to focus research efforts on how to implement this change in field conditions. The test algorithm will need adaptation to incorporate the strength of the test line. A key feasibility issue will be the subjective nature of interpretation of the lines. We used a reference card with photographs and were able to achieve good agreement as evidenced by the kappa levels of 0.79 and 0.85 for KHB. Agreement for STAT-PAK® was less consistent, however the number of weak positives was small. Several authors have reported good agreement between field staff interpretation of the test line strength and that of the reference laboratory [4,22]. However this needs to be evaluated outside of study conditions particularly given the different cadres of staff involved in testing. Bench aids to guide interpretation and a simple training package need to be developed. Finally, to avoid losing individuals to follow up, it will be important to have timely access to confirmation testing as well as good counselling to explain the need for follow up testing.

**Table 8:** **Summary of reports in the literature of performance of RDTs and algorithms with weak positives included and excluded**

Paper	**Algorithm**	**Weak positives**	**N**	**HIV prevalence%**	**Sensitivity (95% CI)**	**Specificity (95% CI)**	**PPV (95% CI)**	**NPV (95% CI)**
Gray, Uganda	D-S-U	Included	639	14.6	97.8	94.1	74.0	99.6
	D-S-U	Excluded	602	14.4	97.7	99.6	97.7	99.6
Klarkowski, DRC	D-U	Included	2728	7.5	100	99.0	89.5	100
					(98.2-100)	(98.6-99.4)	(84.8-93.2)	(99.9-100)
	D-U	Excluded	2711	7.6	100	99.7	96.7	100
					(98.2-100)	(99.4-99.9)	(93.3-98.7)	(99.9-100)
Galiwango, Uganda	D-S-U*	Included	2520	35.2	97.4	99.6	99.3	99.8
					(96.1–98.4)	(99.2–99.9)		
	D-S-U*	Excluded		35.2	97.4	99.8	99.7	99.8
					(96.1–98.4)	(99.5–100)		
	S-D-U**	Included	2520	35.2	99.7	96.9	94.6	99.8
					(99.0–99.9)	(96.0–97.7)		
	S-D-U**	Excluded		35.2	99.7	99.7	99.4	99.8
					(99.0–99.9)	(99.3–99.9)		
	D-S-U**	Included	2520	35.2	97.4	99.7	99.4	98.6
					(96.1–98.4)	(99.3–99.9)		
	D-S-U**	Excluded		35.2	97.3	99.9	99.8	98.6
					(96.0–98.3)	(99.6–100)		
Kagulire, Uganda	D	Included	150	NR	NR	85.2	67.3	NR
							(52.9-79.7)	
	D	Excluded	138	NR	NR	95.1	NR	NR
Kroidl, Tanzania	D (whole blood)	Included	1696	1.5	100	96.8	32.9	100
					(86.7-100)	(95.9-97.6)	(22.8-44.4)	(99.8-100)
	D (whole blood)	Excluded	1646	1.5	100	NR	86.2	NR
	D (plasma)	Included	12916	9.2	100	97.9	82.6	100
					(99.7-100)	(97.6-98.1)	(80.5-84.5)	(99.8-100)
	D(plasma)	Excluded	12692	9.2	100	NR	96.4	NR

S = STAT-PAK, U = Unigold, D = Determine.

DRC = Democratic Republic of Congo.

NR = Not reported.

*Parallel tiebreaker: both Determine and STAT-PAK are done initially, with discordant results resolved by Unigold.

**Serial tiebreaker: first RDT used as a screen; if negative, no further testing. If positive, second test is employed. Discordant results resolved by Unigold.

While our study did not have any false positives without weak positives on one or more RDTs, false positive results are documented to occur with strongly reacting test lines [5,12]. Re-classifying weak positives as inconclusive will not therefore eliminate the risk of false positive reactions.

The strengths of this study are its use of a standard WHO design for evaluation of RDTs, as well as the use of DNA PCR to resolve indeterminate OIC and Western Blot results. The latter avoids misclassification of severely immunosuppressed patients or seroconvertors as false positives. The identification of the weak positive test lines was strengthened by the use of a photo card. A limitation is that we were unable to confirm all of the negative results, and instead tested a random sample of 10% of the negatives. We adjusted for this in the analysis using statistical methods.

In total, 16 individuals were misclassified as HIV positive using the national algorithm. The consequences of receiving a diagnosis of HIV can be devastating to an individual and family. Four of these individuals had CD4 counts less than 350, and 7 had counts less than 500. This suggests that without confirmation testing, these individuals could have been started on ART using new guidelines for ARV initiation. The programmatic consequences of following these individuals in HIV clinics, along with the costs for ancillary laboratory tests are significant [13,23].

Our study suggests that these risks and costs can be eliminated through 3 measures. Firstly, to abandon the tiebreaker regimen in favour of a WHO approved serial or parallel algorithm. Secondly, to consider weak positive results as inconclusive and resolve their status through further testing. And thirdly and most importantly, to introduce a confirmation test to the RDT algorithm using a test that can be performed in peripheral laboratories.

## Conclusion

The risk of false positive HIV diagnosis with the tiebreaker algorithm is significant. False diagnosis of HIV has major consequences for individuals and for health systems. The OIC test improves the diagnostic accuracy of the RDT algorithm and shows good agreement with the gold standard. Weak positive reactions on RDTs are associated with false positive HIV results, and require further testing prior to giving a HIV diagnosis. It is now time to focus research efforts on how best to translate this knowledge into practice at the field level ensuring feasibility in the variety of settings where HIV testing takes place.
